# A Pilot Study of Augmented Intelligence Risk-Based Stratification for Endocrine Surgical Waiting List Prioritisation

**DOI:** 10.7759/cureus.29973

**Published:** 2022-10-06

**Authors:** Lavandan Jegatheeswaran, Neil Tolley

**Affiliations:** 1 Otolaryngology, Imperial College Healthcare NHS Trust, London, GBR

**Keywords:** decision support system, machine learning approaches, waiting list, clinical decision support, artificial intelligence in healthcare

## Abstract

Introduction

The New Deal for Surgery report encouraged using new technology in healthcare to address the 377,689 patients in England awaiting National Health Service (NHS) hospital treatment in July 2022. During the pandemic’s second wave, this pilot study investigated the utility of COMPASS Surgical List Triage (COMPASS SLT; C2-Ai, Cambridge, England), an augmented intelligence-based system, in assisting surgical decision-making on patient prioritisation. Data generated from COMPASS SLT was compared to data from the British Association of Endocrine and Thyroid Surgeons’ (BAETS) and Federation of Surgical Specialties Associations’ (FSSA) prioritisation guidance.

Methods

A cohort of thyroidectomy and parathyroidectomy patients on the surgical waiting list at Imperial College Healthcare NHS Trust, London, United Kingdom, was used. COMPASS SLT calculated individuals’ mortality and significant morbidity risk (risk >2.5%). Significant morbidity risk was set at 2.5% or above following internal model validation, thus reducing the risk of model overfitting occurring with COMPASS SLT. The additional increase in mortality and morbidity due to treatment delay was calculated. Actual treatment time was aligned to the treatment delay (in weeks) experienced by each patient.

Results

Twenty-nine patients, with a median age of 43 years and a waiting time of 18 weeks at the onset of the second wave, were enrolled. Non-statistically significant differences (p=0.937) between the FSSA and BAETS classifications were identified. However, cohort size could promote a type II error. An increase in median mortality and morbidity risk (p<0.001) arising from treatment delay and decisions based on the FSSA and BAETS classifications were identified.

Conclusion

COMPASS SLT can supplement clinical decision-making. An augmented intelligence tool can provide clinicians objectivity and flexibility in prioritising patients, with information on individual morbidity and mortality.

## Introduction

Waiting times for patients scheduled for elective surgery within the National Health Service (NHS) in the United Kingdom (UK) are currently at an all-time high. As of July 2022, there were an estimated 377,689 patients waiting more than 52 weeks for routine operations and procedures, the highest since December 2007 and an increase from 224,205 patients waiting the year prior [1]. There are concerns that this number is an underestimate with there being a “hidden waiting list” of people who have not presented themselves nor been referred to secondary care for surgical treatment [2].

Current guidance on the prioritisation of elective waiting list patients during the pandemic has been reviewed and published on a monthly basis by The Federation of Specialty Surgical Associations (FSSA) [3]. This guidance is written by specialists in the procedures described and sets out the clinician’s view on the relative priorities of conditions at the time the patient is listed. In October 2020, a further two categories have been introduced (P5 and P6); however, these remain largely for administrative purposes [3,4]. For endocrine surgery, the British Association of Endocrine and Thyroid Surgeons (BAETS) developed more detailed prioritisation advice to help clinicians plan their parathyroid, thyroid and adrenal procedures during the pandemic [5]. Both prioritisation guidance split the stratification of these patients into different priority levels (P) from P1a to P6 [3,5]. The breakdown of priority levels is highlighted below:

● Priority Level 1a (P1a): emergency (operation needed within 24 hours to save a life)

● Priority Level 1b (P1b): urgent (operation needed within 72 hours)

● Priority Level 2 (P2): surgery can be safely deferred for up to four weeks - elective surgery with the expectation of a cure

● Priority Level 3 (P3): surgery that can be delayed for up to three months with no predicted negative outcome

●​​​​​​​ Priority Level 4 (P4): surgery that can be delayed for more than three months with no predicted negative outcome

●​​​​​​​ Priority Level 5 (P5): patient wishes to postpone surgery because of COVID-19 concerns

●​​​​​​​ Priority Level 6 (P6): patient wishes to postpone surgery due to non-COVID-19 concerns

Furthermore, the coronavirus disease (COVID-19) pandemic resulted in cancellations of routine operative procedures and redeployment of staff onto acute medical specialities [6], which consequently resulted in the current pre-existing stratification of these patients becoming outdated with patients labelled as P3 being delayed to more than three months [7,8]. More specifically to endocrine surgery, surgical decision-making, case mix and personnel delivering care were significantly affected due to this pandemic [9].

Evidence-based, risk-based stratification scores exist in clinical practice and have been shown to be an effective tool in triaging patients [10,11]. COMPASS Surgical List Triage (COMPASS SLT) is an augmented intelligence evidence-based risk stratification tool designed by Copeland Clinical Artificial intelligence (C2-Ai, Cambridge, England). COMPASS SLT does not predict complications for its patient cohort; instead, it calculates actual observed complication rates initially modelled from an international database of 14 million procedures. Validation for these rates is obtained from a six-monthly refresh whereby complication rates are rechecked on current databases of between four and six million procedures. Given that initial modelling was performed on datasets from the 1990s and early 2000s, the six monthly refreshes have resulted in COMPASS SLT being trained on approximately 200 to 350 million patient records to date. This tool has been validated for use as a safe substitute and screening tool for cardiopulmonary exercise testing (CPET) and mortality risk prediction for low-risk patients (<1.5%) (Oral Presentation: Paterson A, Ramesh R, Kim J, Anderson K. COMPASS surgical risk prediction score: a safe adjunct to Cardiopulmonary Exercise Testing. British Orthopaedic Association Annual Congress; September 23^rd^ 2020). In endocrine surgery, procedures have been validated against the UK Registry of Endocrine and Thyroid Surgery (UKRETS) (Oral Presentation: Ng J, Munroe-Gray T, Palazzo F, Tolley N. UKRETS - A validation study with CRAB, COMPASS and HES Databases. British Association of Endocrine and Thyroid Surgeons 2019 Annual Scientific Meeting; October 3^rd ^2019), a mandatory national registry and audit of endocrine operations performed in the UK [12].

This study was designed to identify discrepancies in the clinical risk grading of patients using FSSA and BAETS guidance. Data were then intended to be compared by additional granularity on patient risk using the COMPASS SLT tool. The study was also designed to assess and quantify the impact and predict the significance of patient treatment delay on morbidity and mortality.

## Materials and methods

Ethical approval was not required by the NHS Health Research Authority decision toolkit.

A cohort of the endocrine surgical waiting list during the second wave of the coronavirus pandemic at Imperial College Healthcare NHS Trust, London, United Kingdom, was used for analysis. Endocrine surgery was chosen, as COMPASS SLT has been previously validated against the UKRETS database (Oral Presentation: Ng J, October 3rd 2019). Patients listed for “Hemithyroidectomy”, “Total Thyroidectomy” and “Parathyroidectomy” were used in the pilot cohort.

The current waiting time was calculated at the time when the patient was first listed following the onset of the second pandemic wave, with 1st February 2021 set as the start date. The listed procedure code was obtained from the Office of Population Censuses and Surveys Classification of Interventions and Procedures (OPCS 4.9), which is a Fundamental Information Standard that is revised on a periodical basis [13].

The COMPASS SLT tool was used to calculate the following parameters for each individual: their overall risk of death, overall risk of complications, a list of all complications with a risk above 2.5% and the overall effect on mortality and morbidity risk, including specific complications, should the surgery be delayed. The significant morbidity risk was set at 2.5% or above following internal model validation. Consequently, this reduces the risk of model overfitting in COMPASS SLT, thus reducing the underestimation of events in low-risk patients and the overestimation of events in high-risk patients [14].

**Figure 1:**
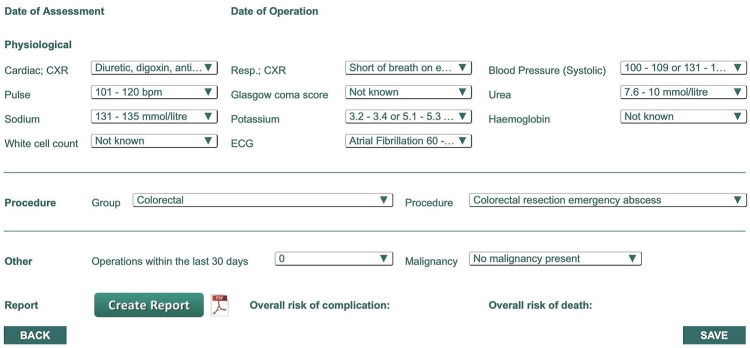
Pre-Operative Physiological Data Required by COMPASS SLT for Output Creation COMPASS SLT: COMPASS Surgical List Triage

The data utilised by COMPASS SLT in order to create its output can be seen in Figure 1, with complication and mortality outputs calculated by the tool seen in Table 1. The delay effect was calculated with the incorporation of the number of weeks that the patient had their operative procedure delayed.

**Table 1:** List of Output Parameters Calculated by COMPASS SLT COMPASS SLT: COMPASS Surgical List Triage

General Complications
Wound infection
Chest infection
Acute renal injury
Wound dehiscence
Cardiac or respiratory problems
Bleeding/excessive bruising
Myocardial infarction
Pulmonary embolus
Deep vein thrombosis
Acute urinary retention
Specific Complications for Procedure (Total Thyroidectomy/Hemi-thyroidectomy/Parathyroidectomy)
Hypocalcaemia
Stridor
Recurrent laryngeal nerve palsy
Overall risk of complication
Overall risk of death

Statistics were performed using SPSS Version 28. To calculate the statistical significance of the operative delay effect, a Wilcoxon signed-rank test was used to account for the non-parametric distribution of data from the matched samples. The same statistical test was also used to compare differences between the FSSA and BAETS prioritisation guidance.

## Results

Demographics

Twenty-nine patients were enrolled for analysis. The median age of this population was 48 years (IQR 14; UQ 57; LQ 43). In this cohort, 23 patients were female and six patients were male. As seen in Table 2, this cohort comprised 16 patients listed for a parathyroidectomy, three patients listed for a hemithyroidectomy and 10 patients listed for a total thyroidectomy. Of the 16 patients listed for a parathyroidectomy, 15 had the primary diagnosis of primary hyperparathyroidism and one patient had the diagnosis of tertiary hyperparathyroidism. With regards to the hemithyroidectomy cohort, two patients had the primary diagnosis of a unilateral goitre and one patient had the primary diagnosis of toxic adenoma. Of the remaining 10 patients that were listed for total thyroidectomy, two were listed due to refractory Graves’ disease non-amenable to medical management, five had the primary diagnosis of bilateral multinodular goitre with indeterminate nodules with Thy3 cytology, and three had bilateral multinodular goitres with compressive symptoms.

**Table 2:** Breakdown of patients by demographics, listed procedure and primary diagnosis

	Number of Patients (n=29)
Demographics	
Age, years (median (LQ, UQ))	48 (43, 57)
Sex	
Male	6
Female	23
Listed Procedure	
Parathyroidectomy	16
Primary Hyperparathyroidism	15
Tertiary Hyperparathyroidism	1
Hemi-thyroidectomy	3
Unilateral Goitre	2
Toxic Adenoma	1
Total Thyroidectomy	10
Refractory Graves’ Disease	2
Bilateral multinodular goitre with indeterminate nodules (Thy3 cytology)	5
Bilateral multinodular goitre with compressive symptoms	3

FSSA and BAETS prioritisation

**Figure 2:**
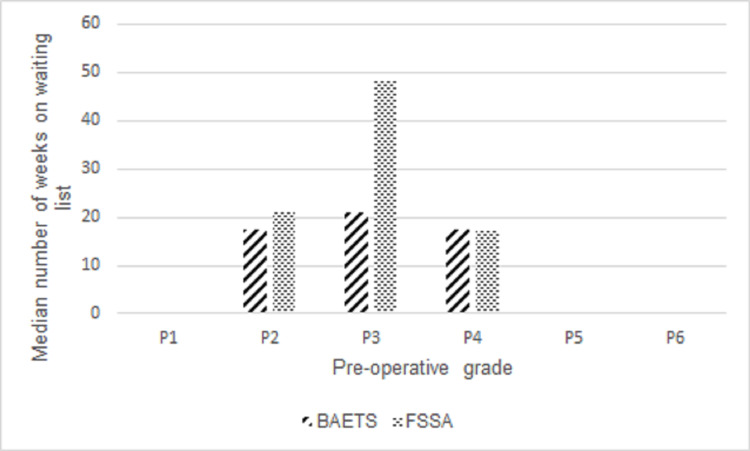
Comparison of Patients Being Classified Under FSSA and BAETS Pre-Operative Guidance on the Imperial College Healthcare NHS Trust Endocrine Patient Waiting List Cohort FSSA: Federation of Surgical Specialty Associations; BAETS: British Association of Endocrine and Thyroid Surgeons; NHS: National Health Service

As seen in Figure 2, under the existing pre-operative prioritisation using FSSA guidance, there were 0 patients classified as P1; one patient classified as P2; one patient classified as P3, and 27 patients classified as P4. When re-classified using the BAETS guidance, there were 0 patients classified as P1, four patients classified as P2, one patient classified as P3 and 24 patients classified as P4. Although clear differences were found between FSSA and BAETS guidance, this did not reach statistical significance, most likely due to the small cohort. This analysis would be underpowered making a type II error more likely (p=0.095).

The median waiting time for this patient cohort was 18 weeks (IQR 11; UQ 25; LQ 14). As seen in Table 3, it was not possible to calculate the significance of the difference in the median number of weeks on the waiting list between the FSSA and BAETS categories for patients listed as P2 and P3. For patients categorised as P4, those listed as P4 under FSSA guidance had a median wait of 17 weeks compared to those listed as P4 under the BAETS guidance (18 weeks); this did not achieve statistical significance (p=0.937).

**Table 3:** Breakdown of Patients by Pre-Operative Grade Classification (BAETS vs FSSA) and Comparison of the Median Wait on the Surgical Waiting List BAETS: British Association of Endocrine and Thyroid Surgeons; FSSA: Federation of Surgical Specialty Associations

Pre-operative grade	Number of Patients (n=29)	Median Number of Weeks on Waiting List	Interquartile Range	Upper Quartile	Lower Quartile	p-value
P2						
BAETS	4	18	15	29	14	NA
FSSA	1	21	0	21	21	
P3						
BAETS	1	21	0	21	21	NA
FSSA	1	48	0	48	48	
P4						
BAETS	24	18	9	23	15	0.937
FSSA	27	17	9	23	14	

COMPASS SLT and calculated morbidity and mortality risk

COMPASS SLT was used to calculate operative risk and pre- and post-treatment delay with the results shown in Table 4. The operative delay increased the operative morbidity and mortality risk in all categories examined.

**Table 4:** Comparison of Morbidity and Mortality Operative Risk (Calculated Using COMPASS SLT) at the Time of Patient Being Listed for Operation and the Impact of the Delay Caused by the Pandemic COMPASS SLT: COMPASS Surgical List Triage COMPASS, C2-Ai, Cambridge, England

Operative Risk (n=29)	Minimum Predicted Risk (%)	Maximum Predicted Risk (%)	Median Predicted Risk (%)	Interquartile Range (Upper Quartile, Lower Quartile) (%)	p-value
Mortality					
At time of listing	0.40	28.30	0.50	0.20 (0.60, 0.40)	<0.001
Delay	0.50	28.30	0.60	0.50 (1.10, 0.50)	
Overall Complication					
At time of listing	5.80	71.90	7.30	2.20 (8.60, 6.40)	<0.001
Delay	6.40	71.90	8.60	5.10 (13.70, 8.60)	
Pneumonia					
At time of listing	2.50	21.00	2.80	0.70 (3.40, 2.70)	<0.001
Delay	2.80	21.00	3.10	1.60 (4.70, 3.10)	
Haemorrhage					
At time of listing	2.00	7.00	2.40	0.50 (2.60, 2.10)	<0.001
Delay	2.00	7.00	2.40	0.40 (2.80, 2.40)	
Myocardial Infarction					
At time of listing	0.30	3.90	0.80	0.40 (1.10, 0.70)	<0.001
Delay	0.30	3.90	1.10	0.70 (1.50, 0.80)	

There was a significant difference in all operative risks studied, which included mortality risk (p<0.001), overall complication risk (<0.001), risk of post-operative pneumonia (p<0.001), risk of post-operative haemorrhage (p<0.001) and risk of post-operative myocardial infarction (p<0.001).

## Discussion

The growing impact of the waiting list endemic

The Kings Fund states that the NHS has one of the most ambitious waiting time standards in the world [15]. It can be argued that these standards can sometimes create perverse incentives by diverting resources and attention to a limited number of services and measures; however, they are an important tool to measure and improve NHS performance. Moreover, they provide accountability to both patients and the public who are major stakeholders in this publicly funded healthcare system [16].

The World Health Organisation (WHO) advised healthcare advisers to avoid neglecting the provision of essential health services, including surgical treatment, during the COVID-19 pandemic [17]. However, in light of the exceptional demand for healthcare resources within the NHS, all elective surgery was to be postponed during the first pandemic wave [8]. The cancellation of elective surgical procedures has resulted in a catastrophic deterioration in an already extant significant waiting list prior to the pandemic. This backlog has implications on both a personal and systemic level, with one study reporting that 30% of patients that had their operative procedures delayed or cancelled during the winter pressures complained of extreme stress and frustration, with another 59% reporting a severe concern about the deterioration of their condition [18] and the effects that this has had on their families. Furthermore, as shown in our data analysis and endorsed by the literature, such a delay has resulted in the worsening of some patients’ primary conditions in addition to increasing their surgical mortality and morbidity with the procedure [7,8].

Multiple factors contribute to the association between treatment delay and the worsening of clinical outcomes. Increasing age, as well as the increased likelihood of surgery being associated with additional comorbidities that are not age-related, such as diabetes, hypertension and obesity, contribute to the worsening of morbidity and mortality risk observed in this pilot cohort. In addition to this, many patients will deteriorate as a consequence of their primary pathology being left untreated. In this pilot study, those that were listed with the primary pathology of primary hyperparathyroidism and left untreated due to treatment delay may be at increased risk of end-organ damage to cardiovascular and bone health, which is well-documented in clinical practice and in literature [19].

In addition, on a systemic level, this negatively impacts trusts’ finances and resource allocation and has a wider impact on training, education and research programmes [20].

The new deal for surgery

The Royal College of Surgeons of England (RCS) acknowledged the negative implications of the pandemic on surgical delivery in their report “New Deal for Surgery” [7]. One of the key recommendations from this report includes: ensuring that all “Integrated Care Systems (“ICSs”) urgently consider what measures can be put in place to support patients facing long waits for surgery, including the best and most efficient use of new technologies to support this” [7].

This pilot study has identified many potential benefits to using a machine learning risk stratification evidence-based tool to assist prioritisation of NHS waiting lists. Clinicians will be able to create a personalised risk assessment based on the patient pre-operative physiological state, which will inform them when making decisions on behalf of their patients. Currently, the prioritisation of patients is formulated using the FSSA and BAETS criteria, and this is based on a Delphi exercise of expert opinion [3,5]. COMPASS SLT adds additional insight to this decision-making process by providing a platform for more objective clinical decision-making using substantial physiological and epidemiological datasets.

The COMPASS SLT tool is quick and easy to use, facilitating a personalised predicted risk calculation of each individual patient. This also permits patients to be informed of the specific risks allied to their proposed elective procedure. Both patients and clinicians can thereby make informed decisions on a shared care basis. At Imperial College Healthcare NHS Trust, this augmented intelligence algorithm permitted a far more precise assessment of an individual patient’s risk and their additional complications resulting from treatment delay, as opposed to a more generic prioritisation based on the type of surgery and pathology only. As a result, a more intelligent and personalised informed consent process occurred.

Cross-referencing additional complication risks to a higher likelihood of an unplanned level 3 admission during the second wave when elective capacity was restricted added to this personalised decision-making process. This new knowledge adds additional benefits during any time of limited NHS elective capacity such as during the winter [18].

NHS England recently released guidance to NHS trusts supporting the benefit of this personalised approach with an aim of assisting the clinical validation of surgical waiting lists and allowing lists to operate effectively [4]. They aim trusts to achieve this by:

● Checking on a patient’s condition and establishing any additional risk factors

● Establishing the patient’s wishes regarding treatment

● Providing good communication between the patient and carer and general practitioner

● Introducing the P5 and P6 categories that allows patients to postpone surgery but remain on the waiting lists

Becoming smarter with augmented intelligence

Evidence-based risk stratification tools are not new to clinical practice, the integration of the Physiological and Operative Severity Score for the enUmeration of Mortality and Morbidity (POSSUM) in emergency surgery patients has resulted in a measurable improvement in both the morbidity and mortality of emergency patients [10,11]. POSSUM was initially devised using logistic regression in UK surgical patients and was initially developed as an adjunct to surgical audit to assess the quality of care in emergency patients [10]. COMPASS SLT is modelled on between 200 and 350 million patient records worldwide, this has reduced algorithm bias that other risk scores may be subject to [21]. The flexibility and scalability of machine learning in comparison to traditional bio-statistical methods renders it extremely versatile, especially in tasks including risk stratification, diagnosis and classification and survival predictions. Furthermore, COMPASS SLT, as with other machine learning tools, continuously learns as more data is inputted. As a consequence, the predictions increase in accuracy with time [22]. This has been proven with other machine learning algorithms used extensively in the creation of risk scores and prognostic scores for patients with heart failure [23].

Using COMPASS SLT to improve clinical governance

Surgeons and hospitals require a gold standard to compare their performance against. COMPASS SLT can facilitate clinicians and hospital trusts to compare their actual performance against the predicted risks calculated for their patients [23]. Audit forms a key pillar of clinical governance within the NHS, the latter of which is seen as important to “safeguard the high standards of care by creating an environment in which excellence in clinical care will flourish” [24].

COMPASS SLT provides an auditable record of how operative prioritisation can be performed. From this small cohort study, there is some evidence that there can be a discrepancy between what patients are initially graded as under the BAETS/FSSA grading guidance compared to what COMPASS SLT establishes the predicted risk being in “reality”. This is quite evident for one patient who was listed as P4 for their total thyroidectomy in January 2020 and consequently waited over 52 weeks for their procedure. However, despite this pre-operative grading, they were deemed to be high-risk according to COMPASS SLT with a mortality and morbidity risk of 28.3% and 71.9% respectively.

This is not a novel phenomenon. In relation to metabolic equivalents (MET) for myocardial ischaemia, one study showed that predictive METs calculated using pre-operative history-taking tended to be underestimated when in comparison to predictive METs calculated using a cardiac stress test [25]. The presence of additional objective data may reduce the impact of cognitive bias on decision-making and potentially reduce the likelihood of human error being incorporated into clinical decision-making [26]. With the huge potential that augmented intelligence can bring to this area, it is imperative that clinicians and individuals involved in healthcare management understand, embrace and integrate the rapid changes occurring within this field. This will help facilitate effective, economic and safe healthcare delivery in the future [27].

Limitations

The authors acknowledge that limitations are incumbent on a small pilot cohort study and the conclusions made. The small cohort and the use of the endocrine surgical waiting list sample may not be representative of how COMPASS SLT can relate to other specialities.

Future work to analyse and validate the FSSA, BAETS and COMPASS SLT systems will require a more comprehensive study, which in turn would require ethical approval to validate their outcomes [28]. It is important to emphasise that COMPASS SLT was not intended to replace the FSSA and BAETS prioritisation methodology but to provide additional granularity on individual patient risk and to facilitate a more informed approach to surgical waiting list management.

## Conclusions

The NHS has an all-time record high of patients on the waiting list for treatment. Exploring novel technology, such as COMPASS SLT, can supplement clinical decision-making. An augmented intelligence tool can provide clinicians objectivity and flexibility in prioritising patients, with information on individual morbidity and mortality. Further comprehensive studies need to be designed to assess the impact of COMPASS SLT in clinical decision-making across different specialities. Secondary studies on this topic will help assess the impact of treatment delay costs on patients and the NHS.
